# Case Report: Ectopic pancreas mimicking gastrointestinal subepithelial tumors: diagnostic challenges in two surgically confirmed sibling cases

**DOI:** 10.3389/fmed.2026.1903884

**Published:** 2026-07-17

**Authors:** Xiaoxia Li, Juanxian Gu, Xianghua Zhou, Liangliang Wang, Saiqi Zhang, Xiao Huang

**Affiliations:** 1 Department of Intensive Care Medicine, Haining People’s Hospital, Jiaxing, China; 2 Department of Pathology, Haining People’s Hospital, Jiaxing, China; 3 Department of Radiology, Haining People’s Hospital, Jiaxing, China; 4 The Second School of Clinical Medicine, Zhejiang Chinese Medical University, Hangzhou, China; 5 Department of Neurosurgery, Haining People’s Hospital, Jiaxing, China

**Keywords:** case report, ectopic pancreas, gastrointestinal stromal tumor, gastrointestinal subepithelial tumor, Heinrich classification, heterotopic pancreas, sibling cases

## Abstract

**Background:**

Ectopic pancreas is a congenital developmental anomaly that may occur in the stomach, duodenum, jejunum, and other gastrointestinal sites. Because its clinical, laboratory, and imaging features are often nonspecific, it may mimic gastrointestinal subepithelial tumors, particularly when presenting as a submucosal, intramural, or wall-based mass. Gastrointestinal stromal tumor (GIST) is often an important preoperative differential diagnosis, but it is not the only diagnostic consideration.

**Case presentation:**

We report two biological sisters with pathologically confirmed ectopic pancreas. Case 1 was the younger sister. She was asymptomatic and was found to have a gastric submucosal lesion and an enhancing small intestinal nodule during a routine health check-up. Gastroscopy, endoscopic ultrasonography, and contrast-enhanced computed tomography suggested a gastrointestinal subepithelial tumor, with GIST considered as the leading preoperative diagnosis, and both lesions were surgically resected. Histopathology confirmed ectopic pancreas at both sites. Case 2 was the elder sister. She presented with right upper abdominal pain radiating to the back, low-grade fever, elevated C-reactive protein, and a proximal jejunal wall-based mass on multimodal imaging. GIST could not be excluded before surgery. Laparoscopic resection was performed, and histopathology confirmed ectopic pancreas with local inflammatory changes.

In both cases, the resected lesions showed mature pancreatic acini, ducts, and islets, consistent with Heinrich type I and Gaspar-Fuentes type I ectopic pancreas. Case 1 showed no evidence of recurrence by April 2026. Case 2 had no obvious abdominal symptoms during short-term follow-up until April 2026, but long-term recurrence, symptom control, and postoperative complications could not be fully assessed.

**Conclusion:**

Ectopic pancreas may present as gastrointestinal wall-based tumor-like lesions and closely mimic GIST or other gastrointestinal subepithelial tumors on preoperative imaging, particularly when typical features such as central umbilication or duct-like structures are absent. Histopathological examination remains essential for definitive diagnosis. Although both patients were biological sisters, the current evidence is insufficient to support hereditary origin or definite familial clustering.

## Introduction

Ectopic pancreas, also known as heterotopic pancreas or pancreatic heterotopia, refers to pancreatic tissue located outside the normal anatomical site of the pancreas, without direct anatomical, vascular, or ductal continuity with the orthotopic pancreas (1, 2). It is generally considered a congenital developmental anomaly. It most commonly occurs in the upper gastrointestinal tract, including the stomach, duodenum, and jejunum. Most patients are asymptomatic, and the condition is often detected incidentally during health examination, endoscopy, imaging, or surgery (1, 2). A small proportion of patients may present with abdominal pain, vomiting, gastrointestinal bleeding, or other symptoms caused by inflammation, bleeding, obstruction, or mass effect (2, 3).

Preoperative diagnosis of ectopic pancreas is difficult. This is especially true when the lesion presents as a submucosal, intramural, or wall-based tumor-like mass in the gastrointestinal tract. In such cases, it often needs to be distinguished from gastrointestinal stromal tumor (GIST), neuroendocrine tumor, leiomyoma, lipoma, and other mesenchymal tumors (2, 4, 5).

Among these differential diagnoses, GIST is often the leading clinical and radiological consideration because it commonly presents as an enhancing gastrointestinal wall-based or subepithelial mass and may require surgical management. However, GIST is not the only diagnostic possibility. Neuroendocrine tumor, leiomyoma, lipoma, inflammatory lesions, and other mesenchymal tumors may show overlapping clinical and radiological features. Gastric ectopic pancreas may sometimes show central umbilication, an umbilicus-like opening, or a duct-like structure, but these findings are not always present (6). Small intestinal ectopic pancreas usually lacks typical endoscopic and imaging features and is therefore more likely to be misdiagnosed as a neoplastic lesion before surgery (2, 7).

We report two biological sisters who were both confirmed to have ectopic pancreas by postoperative histopathology. Case 1 refers to the younger sister, who had lesions involving both the stomach and the small intestine. Case 2 refers to the elder sister, who presented with a symptomatic lesion in the proximal jejunum. In both cases, GIST was considered preoperatively, but other gastrointestinal subepithelial lesions were also relevant differential diagnoses. This report highlights the diagnostic challenge of ectopic pancreas presenting as solitary or multiple gastrointestinal wall-based lesions and emphasizes the role of histopathological confirmation. The sister relationship is presented as an observed clinical context rather than evidence of hereditary susceptibility or definite familial clustering.

## Case presentation

Two biological sisters were included in this report. Both patients presented with gastrointestinal tumor-like lesions, and GIST was considered the leading preoperative differential diagnosis. Other subepithelial lesions, including neuroendocrine tumor, leiomyoma, lipoma, inflammatory lesions, and ectopic pancreas, were also considered in the differential diagnosis according to lesion location, enhancement pattern, clinical presentation, and available endoscopic or imaging features. The final diagnosis was established by postoperative histopathology. A brief clinical timeline of the two patients is summarized in Table 1.

**TABLE 1:** Clinical timeline of the two patients.

Time point	Case 1	Case 2
Initial presentation	2018, routine health check-up; asymptomatic	January 23, 2026; right upper abdominal pain radiating to the back with low-grade fever
Initial evaluation	Gastroscopy and EUS showed a gastric submucosal lesion	Laboratory tests showed elevated CRP; abdominal CT showed focal small intestinal wall thickening
Imaging impression	Gastric and small intestinal lesions; GIST was considered	Proximal jejunal wall-based lesion on CT, MRI, and PET/CT; GIST was considered
Surgery	December 20, 2018	February 12, 2026
Pathology	Ectopic pancreas in both gastric and small intestinal lesions; Heinrich type I / Gaspar-Fuentes type I	Ectopic pancreas with fat necrosis and inflammatory reaction; Heinrich type I / Gaspar-Fuentes type I
Follow-up	No evidence of recurrence by April 2026	No obvious abdominal symptoms during short-term follow-up until April 2026; long-term recurrence and delayed postoperative complications could not be assessed.

CRP, C-reactive protein; CT, computed tomography; EUS, endoscopic ultrasonography; GIST, gastrointestinal stromal tumor; MRI, magnetic resonance imaging; PET/CT, positron emission tomography/computed tomography.

### Case 1

**FIGURE 1:**
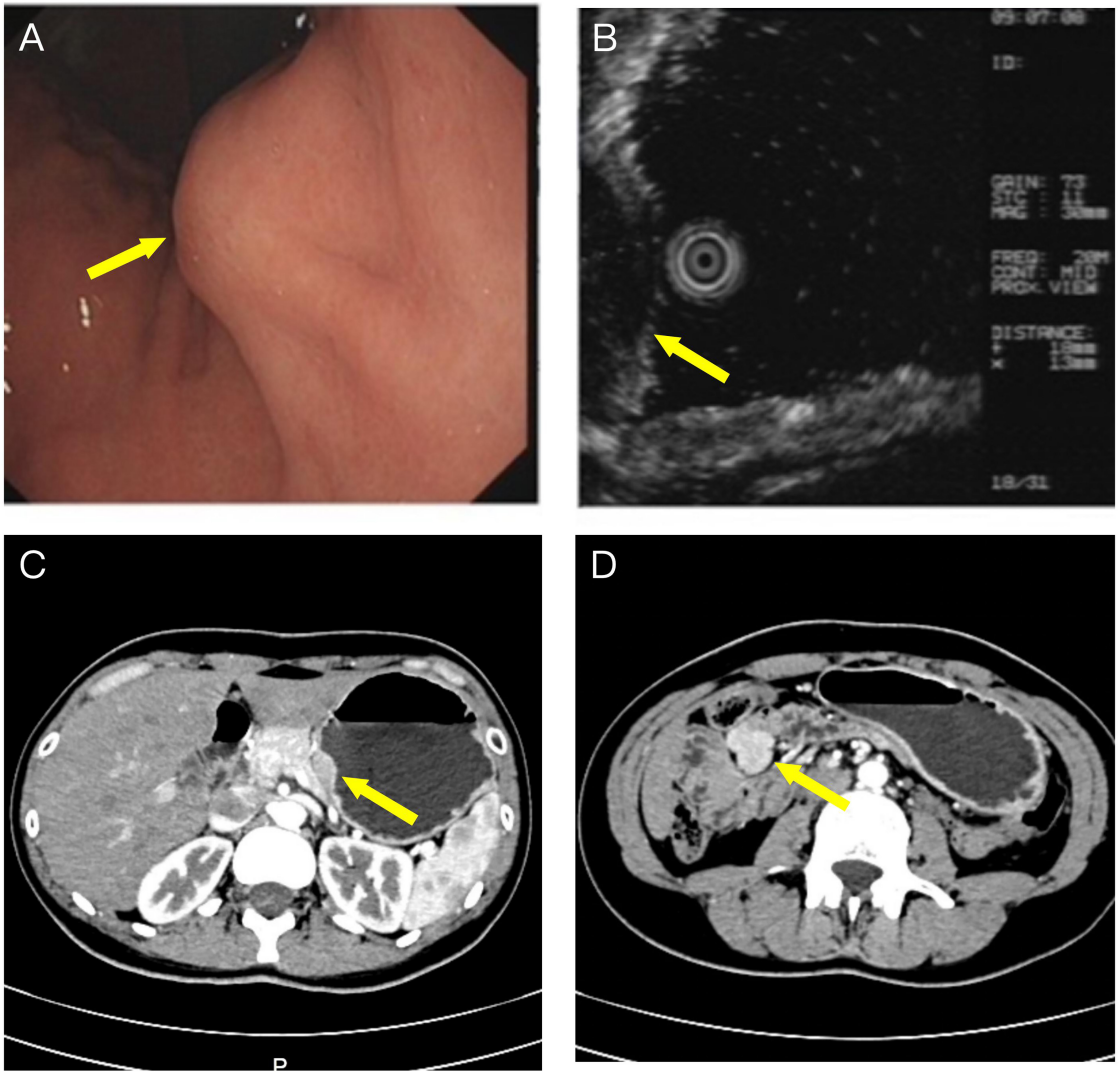
Preoperative endoscopic and imaging findings in Case 1. **(A)** Gastroscopy revealed a submucosal protruding lesion in the gastric body with a smooth surface. **(B)** Endoscopic ultrasonography demonstrated a hypoechoic lesion measuring approximately 1.8 × 1.3 cm arising from the muscularis propria. **(C)** Contrast-enhanced computed tomography (CT) showed a markedly enhancing nodule measuring approximately 16–17 mm along the lesser curvature of the stomach. **(D)** Contrast-enhanced CT revealed an approximately 10-mm enhancing nodule in the small intestine. Arrows indicate the lesions.

The patient was the younger sister, a 24-year-old woman who underwent a routine health check-up in 2018 despite having no abdominal symptoms. Screening gastroscopy during this check-up showed a submucosal lesion in the gastric body. She had no obvious abdominal pain, abdominal distension, nausea, vomiting, or other discomfort at that time. Further endoscopic ultrasonography (EUS) showed a hypoechoic mass arising from the muscularis propria. The layers of the gastric wall were clear and intact. Contrast-enhanced computed tomography (CT) showed a markedly enhanced nodule along the lesser curvature of the stomach. Another enhanced nodule was also found in the small intestine. Based on the lesion locations and enhancement patterns, a gastrointestinal subepithelial tumor was suspected, with GIST considered as the leading preoperative diagnosis (Figure 1).

This preoperative impression was mainly based on the gastric lesion arising from the muscularis propria, the marked enhancement of the gastric nodule, and the presence of an additional enhancing small intestinal nodule. A neuroendocrine tumor was considered less likely because there were no specific clinical manifestations or biochemical findings suggesting a functioning neuroendocrine tumor, although a nonfunctioning lesion could not be excluded by imaging alone. Leiomyoma or other mesenchymal tumors were also considered, but the presence of two enhancing lesions at different gastrointestinal sites made definitive preoperative classification difficult. Ectopic pancreas was a possible differential diagnosis, but typical features such as central umbilication, a duct-like structure, or enhancement clearly similar to the orthotopic pancreas were not identified.

Surgery was selected instead of surveillance because GIST could not be excluded, the gastric lesion appeared to arise from the muscularis propria, and an additional enhancing small intestinal lesion was present. Routine endoscopic biopsy was considered unlikely to provide a reliable diagnosis for a muscularis propria-based lesion. Therefore, surgical resection was performed for both diagnostic confirmation and definitive treatment.

**FIGURE 2:**
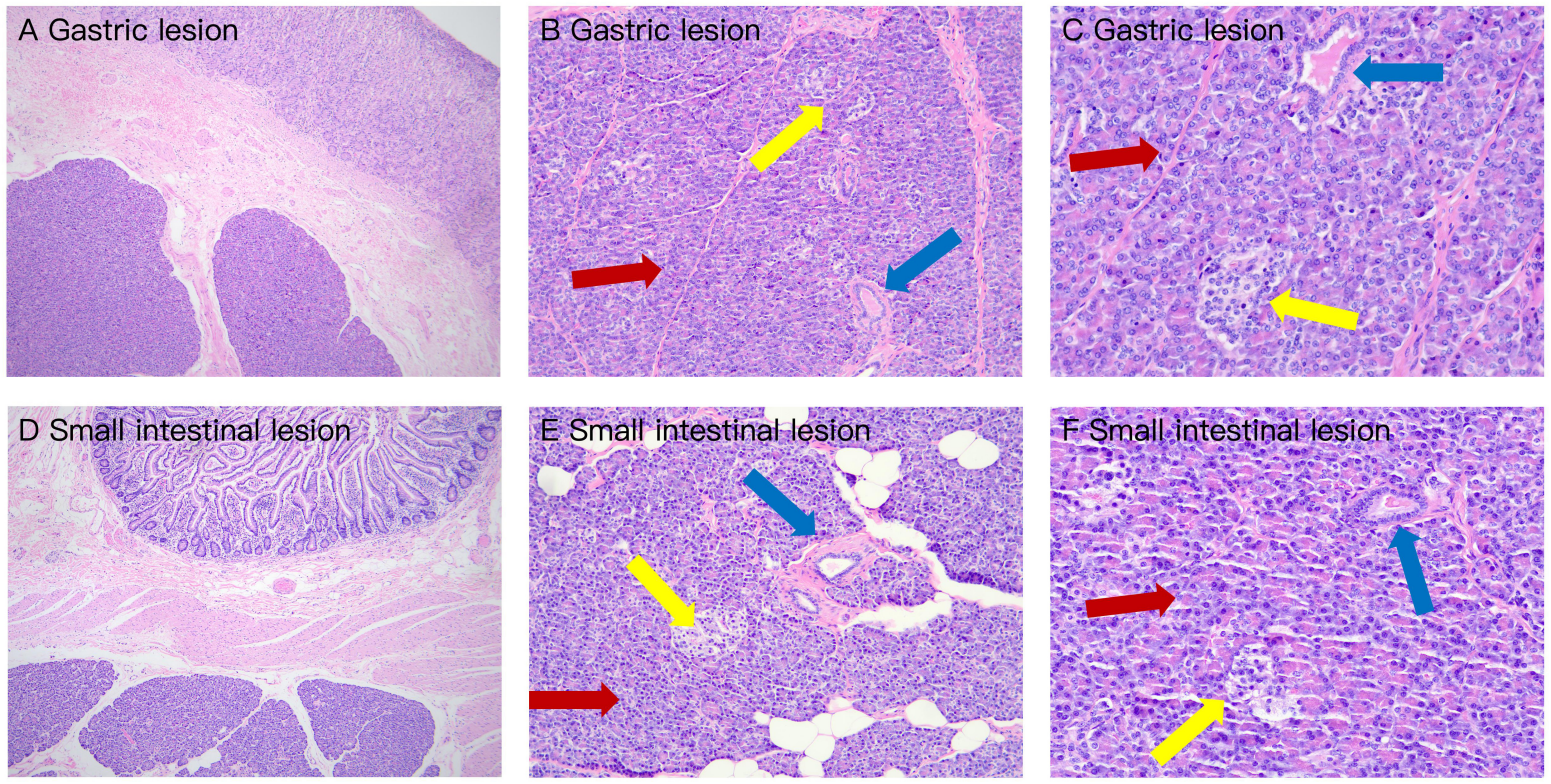
Histopathological findings of the gastric and small intestinal lesions in Case 1. **(A–C)** Histopathological findings of the gastric lesion. **(A)** Low-power view demonstrating ectopic pancreatic tissue located within the gastric wall beneath the mucosa. **(B)** Medium-power view showing lobulated pancreatic tissue composed of pancreatic acini, ductal structures, and scattered islet-like cell clusters, with fibrous septa separating the lobules. **(C)** High-power view demonstrating pancreatic acini, ductal structures, and scattered islet-like cell clusters. **(D–F)** Histopathological findings of the small intestinal lesion. **(D)** Low-power view demonstrating ectopic pancreatic tissue located within the intestinal wall beneath the mucosa. **(E)** Medium-power view showing well-developed pancreatic tissue composed predominantly of pancreatic acini, with scattered ductal structures and islet-like cell clusters. **(F)** High-power view demonstrating pancreatic acini, ductal structures, and scattered islet-like cell clusters. Red arrows indicate pancreatic acini; blue arrows indicate ductal structures; yellow arrows indicate islet-like cell clusters. Both lesions were consistent with Heinrich type I and Gaspar-Fuentes type I ectopic pancreas. Hematoxylin and eosin (HE) staining. Original magnifications: A and D, × 40; B and E, × 100; C and F, × 200.

On December 20, 2018, the patient underwent laparoscopic and endoscopic resection of the gastric body lesion under general anesthesia. Laparoscopic resection of the small intestinal lesion was performed during the same operation. Postoperative histopathology confirmed ectopic pancreas in both the gastric and small intestinal lesions. Further pathological review showed mature pancreatic parenchymal structures within the lesions. Pancreatic acini, ducts, and islets were all well differentiated. The proportions of these three components were close to those seen in normal pancreatic tissue. These findings were consistent with Heinrich type I and Gaspar-Fuentes type I ectopic pancreas, corresponding to mature-type ectopic pancreas (Figure 2). No histological features suggestive of GIST were identified.

The small intestinal specimen measured approximately 3.0 × 2.5 cm, with negative resection margins. Preoperative CT showed that the enhanced small intestinal nodule was approximately 10 mm, whereas the postoperative specimen was larger on gross examination. This difference may be related to different measurement methods. Imaging mainly reflected the more obviously enhanced part of the lesion, while gross pathological measurement was based on the entire resected specimen. The gastric lesion measured approximately 2.2 × 2.0 × 1.5 cm. One resection margin was close to the lesion, while the other margin was negative.

The patient recovered well after surgery. During 2019–2020, the patient experienced several episodes of incomplete small bowel obstruction, which resolved after conservative treatment. The relationship between these episodes and the previous surgery remained uncertain. No evidence of lesion recurrence was found during subsequent follow-up. At follow-up in April 2026, the patient had no obvious abdominal symptoms. Repeat abdominal CT and gastroscopy showed no obvious abnormalities.

### Case 2

The patient was the elder sister, a 41-year-old woman who presented in January 2026. On January 23, 2026, she developed abdominal pain without an obvious trigger. The pain was mainly located in the right upper abdomen and radiated to the back. She also had low-grade fever, with a maximum temperature of 37.8 °C. During the disease course, she experienced intermittent chest tightness. Electrocardiography and myocardial enzyme tests showed no abnormalities.

Laboratory tests on January 25, 2026, showed a C-reactive protein (CRP) level of 24.96 mg/L and a white blood cell count of 9 × 10^9^/L. The patient received intravenous ceftriaxone at a dose of 2.0 g as anti-infective treatment. After 5 days of treatment, the abdominal pain did not improve significantly. Abdominal CT showed focal thickening of the small intestinal wall with outward protrusion. Inflammatory exudative changes were observed around the lesion.

**FIGURE 3:**
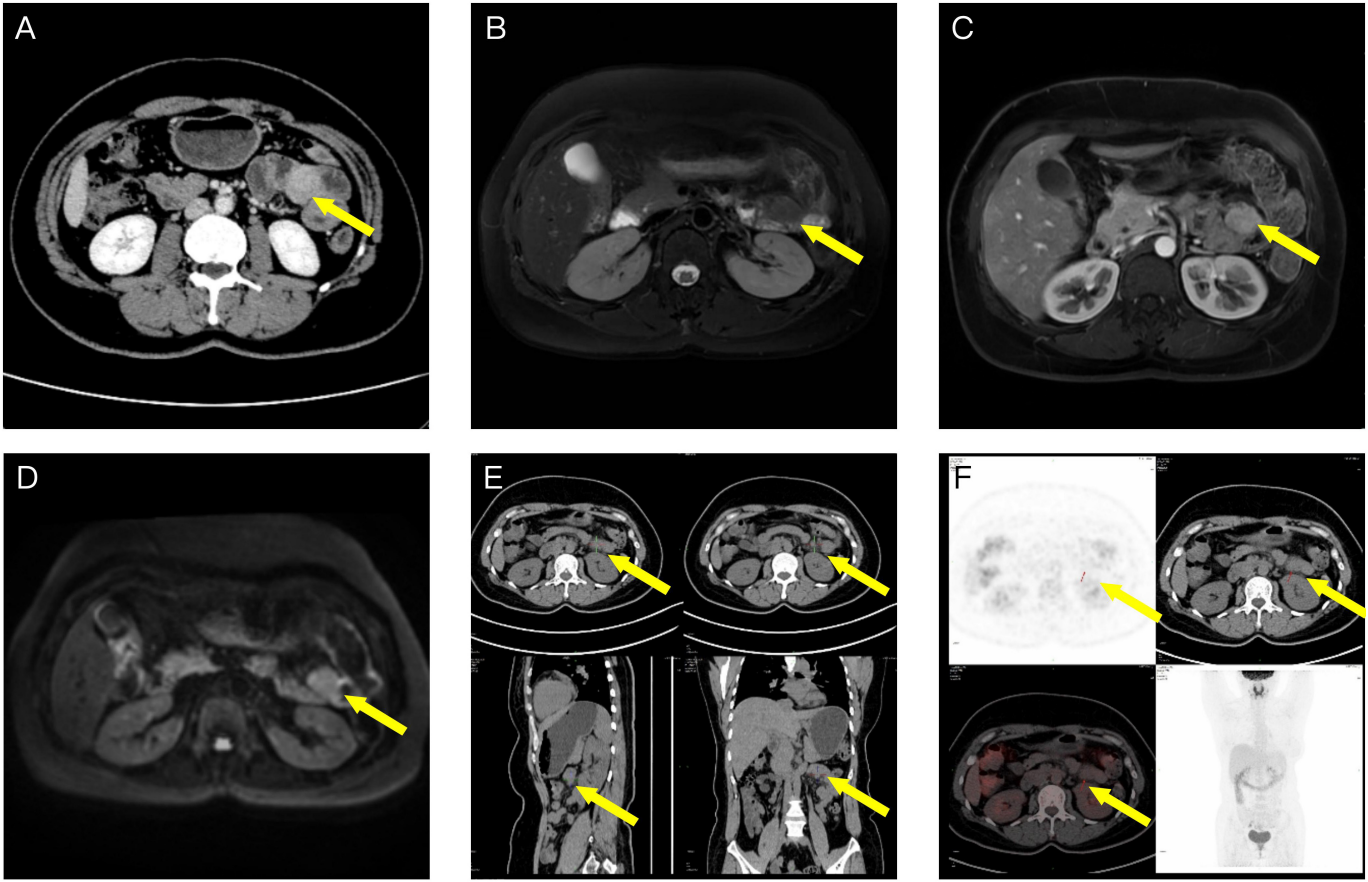
Preoperative imaging findings in Case 2. **(A)** Contrast-enhanced computed tomography (CT) revealed a nodular slightly hyperdense lesion on the mesenteric side of the proximal jejunum in the left mid-abdomen, measuring approximately 2.7 × 2.4 cm, with heterogeneous enhancement and an indistinct boundary from the adjacent bowel wall. **(B)** Fat-suppressed T2-weighted imaging (T2WI-FS) showed a slightly hyperintense lesion in the proximal jejunum. **(C)** Contrast-enhanced T1-weighted imaging (CE-T1WI) demonstrated enhancement of the lesion. **(D)** Diffusion-weighted imaging (DWI) showed mildly increased signal intensity in part of the lesion. **(E,F)** Positron emission tomography/computed tomography (PET/CT) demonstrated a soft tissue density lesion on the mesenteric side of the proximal jejunum measuring approximately 1.9 × 1.7 cm, with mildly increased fluorodeoxyglucose uptake and a maximum standardized uptake value (SUVmax) of 2.6. Arrows indicate the lesions.

Because the symptoms persisted, contrast-enhanced CT, contrast-enhanced magnetic resonance imaging (MRI), and positron emission tomography/computed tomography (PET/CT) were performed. Multimodal imaging showed a nodular lesion on the mesenteric side of the proximal jejunum. The lesion was closely related to the adjacent intestinal wall and showed heterogeneous enhancement after contrast administration. Contrast-enhanced MRI showed soft tissue mass-like signal features. PET/CT showed mildly increased fluorodeoxyglucose (FDG) uptake in the lesion (Figure 3). This mild FDG uptake did not strongly support malignancy, but it was not specific enough to exclude GIST before surgery. Based on the overall imaging findings, GIST was considered as the leading preoperative diagnosis. This impression was mainly based on the wall-based location of the lesion, its close relationship with the adjacent jejunal wall, and its heterogeneous contrast enhancement.

The differential diagnosis also included neuroendocrine tumor, leiomyoma, inflammatory mass, and ectopic pancreas. A neuroendocrine tumor was considered because small intestinal neuroendocrine tumors may present as enhancing wall-based lesions; however, the absence of specific endocrine symptoms and the mild FDG uptake did not strongly support this diagnosis. Leiomyoma and other mesenchymal tumors could not be excluded because of the wall-based growth pattern. Inflammatory lesions were also considered because of abdominal pain, elevated CRP, and surrounding inflammatory exudation. Ectopic pancreas was also considered, but the lesion lacked typical preoperative features such as central umbilication or a duct-like structure.

Surgery was chosen because the patient had persistent abdominal pain despite anti-infective treatment, inflammatory changes were present around the lesion, and GIST or another neoplastic lesion could not be excluded by multimodal imaging. Therefore, laparoscopic resection was performed for both therapeutic management and definitive pathological diagnosis.

**FIGURE 4:**
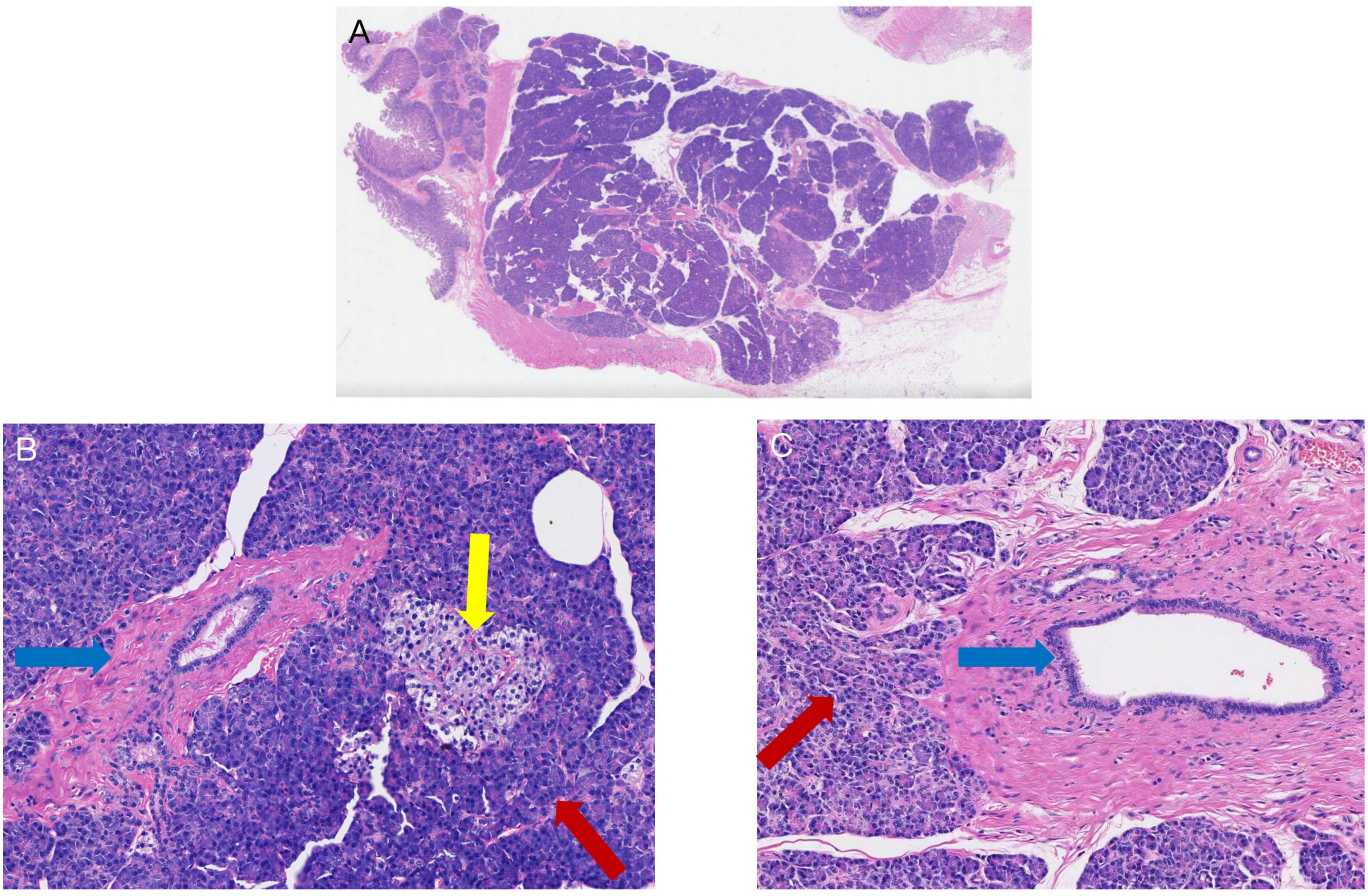
Histopathological findings of the small intestinal lesion in Case 2. **(A)** Representative low-power field selected from whole-slide imaging, demonstrating lobulated ectopic pancreatic tissue located within the intestinal wall beneath the mucosa. **(B)** Representative medium-power field showing mature pancreatic tissue composed of pancreatic acini, ductal structures, and scattered islet-like cell clusters. **(C)** Representative high-power field demonstrating well-developed ductal structures, pancreatic acini, and scattered islet-like cell clusters. Red arrows indicate pancreatic acini; blue arrows indicate ductal structures; yellow arrows indicate islet-like cell clusters. The findings were consistent with Heinrich type I and Gaspar-Fuentes type I ectopic pancreas. Hematoxylin and eosin (HE) staining. Images were selected from scanned whole-slide images; conventional optical magnifications were not available.

On February 12, 2026, the patient underwent laparoscopic resection of the small intestinal lesion under general anesthesia. Postoperative histopathology showed ectopic pancreatic tissue. The lesion measured approximately 3.2 × 2.2 × 2.0 cm. Pathological review showed mature pancreatic parenchymal structures within the lesion. Pancreatic acini, ducts, and islets were all well differentiated. The proportions of these three components were close to those seen in normal pancreatic tissue. These findings were consistent with Heinrich type I and Gaspar-Fuentes type I ectopic pancreas, corresponding to mature-type ectopic pancreas. Fat necrosis nodules were observed around the lesion, with aggregation of foamy histiocytes and a multinucleated giant cell reaction. No obvious abnormality was found at the small intestinal resection margins (Figure 4). No spindle-cell proliferation or other morphological features supporting GIST were observed on histopathological examination.

The patient recovered uneventfully after surgery. During follow-up until April 2026, she had no obvious abdominal symptoms. Because the follow-up period was short, long-term symptom control, recurrence-free status, and late postoperative complications could not be fully evaluated.

In both patients, preoperative tumor markers, serum amylase, serum lipase, liver function tests, and renal function tests were within the institutional reference ranges.

## Discussion

The present cases demonstrate two clinically important aspects of ectopic pancreas. First, ectopic pancreas may closely resemble gastrointestinal subepithelial tumors on preoperative evaluation. In Case 1, the gastric and small intestinal lesions presented as separate mass-like lesions. In Case 2, the proximal jejunal lesion showed imaging features that made GIST the leading preoperative consideration. However, the differential diagnosis should not be limited to GIST alone. Neuroendocrine tumors, leiomyomas, lipomas, inflammatory lesions, and other mesenchymal tumors may show overlapping clinical and radiological features. Therefore, ectopic pancreas should be considered when evaluating gastrointestinal subepithelial lesions, particularly when imaging findings are atypical or when characteristic features of GIST are not definitive. Second, the occurrence of ectopic pancreas in two biological sisters is unusual and makes the cases clinically noteworthy. Nevertheless, this observation should be interpreted cautiously. The present report includes only two surgically confirmed cases, and no genetic testing or systematic family assessment was performed. Therefore, a hereditary association or definite familial clustering cannot be established. The sister relationship should be described only as an observed clinical context rather than evidence of hereditary susceptibility.

Ectopic pancreas is generally regarded as a congenital embryological anomaly, although its exact pathogenesis remains unclear. Several embryological theories have been proposed, including abnormal separation or migration of pancreatic tissue during foregut rotation and gastrointestinal development (2, 3, 8, 9). This mechanism may explain why ectopic pancreas can occur in the stomach, duodenum, jejunum, and other gastrointestinal sites. It may also explain the involvement of both the stomach and small intestine in Case 1. However, this developmental mechanism is not equivalent to a genetic disorder.

Histopathological examination remains the basis for the definitive diagnosis of ectopic pancreas. The diagnosis relies on the identification of pancreatic tissue components within the lesion, including pancreatic acini, ducts, and/or islets. According to the Heinrich classification, type I ectopic pancreas contains acini, ducts, and islets and therefore closely resembles normal pancreatic tissue; type II contains acini and ducts but lacks islets; and type III is predominantly composed of duct-like structures (3, 10). The Gaspar-Fuentes classification later modified this system, but type I similarly refers to complete ectopic pancreas containing acini, ducts, and islets (10). In the present report, the gastric lesion and small intestinal lesion in Case 1, as well as the proximal jejunal lesion in Case 2, all showed well-differentiated pancreatic acini, ducts, and islets, supporting the diagnosis of Heinrich type I and Gaspar-Fuentes type I ectopic pancreas. In Case 2, surrounding fat necrosis, foamy histiocyte aggregation, and multinucleated giant cell reaction suggested a local inflammatory reaction, which may explain the patient’s abdominal pain, low-grade fever, and elevated inflammatory markers.

Preoperative imaging differentiation was a major diagnostic challenge in these cases. Gastrointestinal stromal tumors (GISTs) usually arise from the muscularis propria and may show intramural, endophytic, or exophytic growth (11). On contrast-enhanced CT or MRI, GISTs often appear as well-defined gastrointestinal wall-based masses, with homogeneous enhancement in smaller lesions or heterogeneous enhancement in larger lesions because of necrosis, hemorrhage, or cystic degeneration (12). In Case 1, the gastric lesion arose from the muscularis propria and showed marked enhancement, and an additional enhanced small intestinal nodule was also present. In Case 2, the proximal jejunal lesion was closely related to the adjacent bowel wall and showed tumor-like features on contrast-enhanced CT, MRI, and PET/CT. These findings made GIST a reasonable preoperative consideration in both patients. However, they were not specific enough to exclude ectopic pancreas. The absence of typical features of ectopic pancreas, such as central umbilication, a duct-like structure, or enhancement clearly similar to the orthotopic pancreas, further increased the diagnostic difficulty.

When weighing the differential diagnosis before surgery, GIST was prioritized because both cases showed enhancing gastrointestinal wall-based lesions, and the gastric lesion in Case 1 appeared to arise from the muscularis propria. Neuroendocrine tumor was considered because it may also present as an enhancing gastrointestinal lesion, but there were no specific endocrine symptoms or biochemical abnormalities. Leiomyoma and other mesenchymal tumors remained possible because imaging alone cannot reliably distinguish these entities from GIST. In Case 2, an inflammatory mass was also considered because of abdominal pain, elevated CRP, and surrounding exudative changes. However, the persistent symptoms and the inability to exclude neoplasia supported surgical resection. These considerations illustrate why definitive diagnosis was not possible before histopathological examination.

Ectopic pancreas, particularly gastric ectopic pancreas, may show central umbilication or an umbilicus-like opening on endoscopy, and duct-like structures may be identified on endoscopic ultrasound or magnetic resonance cholangiopancreatography (1, 2). On contrast-enhanced CT or MRI, ectopic pancreas may show enhancement similar to that of the orthotopic pancreas, although this finding is not consistently present (2, 13). In the present cases, no definite central umbilication or duct-like structure was observed. This was particularly true in Case 2, in which the lesion was located on the mesenteric side of the small intestine and lacked typical endoscopic or imaging features (2, 7). Therefore, ectopic pancreas can be easily confused with GIST, neuroendocrine tumor, or other mesenchymal tumors before surgery. For gastrointestinal submucosal, intramural, or wall-based tumor-like lesions that are suspected to be GISTs on imaging but lack pathognomonic features, ectopic pancreas should be included in the differential diagnosis (2, 4). A definitive diagnosis still relies on histopathological examination (2, 14).

In terms of treatment, follow-up observation may be considered for asymptomatic and small ectopic pancreas when there are no concerning clinical, endoscopic, or imaging features (8, 15). However, resection remains a reasonable option for patients with symptoms, lesion enlargement, diagnostic uncertainty, suspected neoplastic change, or complications such as bleeding, obstruction, or inflammation (3, 10). In the present report, both patients underwent surgery because GIST could not be excluded before the operation. For Case 1, although the patient was asymptomatic, surgery was selected because the gastric lesion arose from the muscularis propria, an additional enhancing small intestinal lesion was present, and preoperative imaging could not reliably exclude GIST or other neoplastic lesions. For Case 2, surgery was selected because of persistent abdominal pain, inflammatory changes around the lesion, and diagnostic uncertainty after multimodal imaging. Surgery not only removed the lesions but also provided the final diagnosis. Case 1 showed no evidence of recurrence during subsequent follow-up, although she had several episodes of incomplete small bowel obstruction after surgery that resolved with conservative treatment. The relationship between these episodes and the previous surgery or ectopic pancreatic lesions remained uncertain. Case 2 had only completed short-term postoperative follow-up by April 2026 and reported no obvious discomfort during this period. Therefore, no conclusion can be made regarding long-term recurrence-free status, durable symptom control, or delayed postoperative complications, especially in Case 2.

This study has several limitations. First, the number of cases is limited, with only two pathologically confirmed cases of ectopic pancreas. Second, although all lesions were classified as Heinrich type I and Gaspar-Fuentes type I, this remains a case report and cannot assess the relationship between pathological subtype and clinical manifestations. Third, genetic testing and systematic family assessment were not performed. Therefore, we cannot evaluate potential genetic susceptibility or prove definite familial clustering. Finally, the follow-up period for Case 2 was short and only reflects the early postoperative course. Long-term recurrence, symptom control, and delayed postoperative complications cannot be fully assessed. Future studies with more cases, complete pathological data, and longer follow-up may help clarify the clinical significance of similar cases.

## Conclusion

Ectopic pancreas may present as solitary or multiple gastrointestinal wall-based tumor-like lesions and closely mimic GIST or other gastrointestinal subepithelial tumors before surgery. Histopathological examination remains essential for definitive diagnosis. Although the two patients in this report were biological sisters, the current evidence is insufficient to support hereditary origin or definite familial clustering. Ectopic pancreas should be considered in the differential diagnosis of gastrointestinal wall-based lesions suspected to be GIST but lacking specific imaging features. For patients with diagnostic uncertainty, persistent symptoms, suspected neoplastic potential, or multiple enhancing lesions, surgical resection may provide both definitive treatment and pathological confirmation.

## Data Availability

The original contributions presented in this study are included in the article/supplementary material, further inquiries can be directed to the corresponding author.
